# Supplementary material for: Association of Perceived Neighbourhood Walkability with Self-Reported Physical Activity and Body Mass Index in South African Adolescents
Source: Int J Environ Res Public Health. 2023 Jan 30;20(3):2449. doi: 10.3390/ijerph20032449 (PMC9916213; doi:10.3390/ijerph20032449)
Supplement: Supplementary file 1 [file ijerph-2159501-supplementary.pdf › ijerph-2159501-supplementary.pdf]

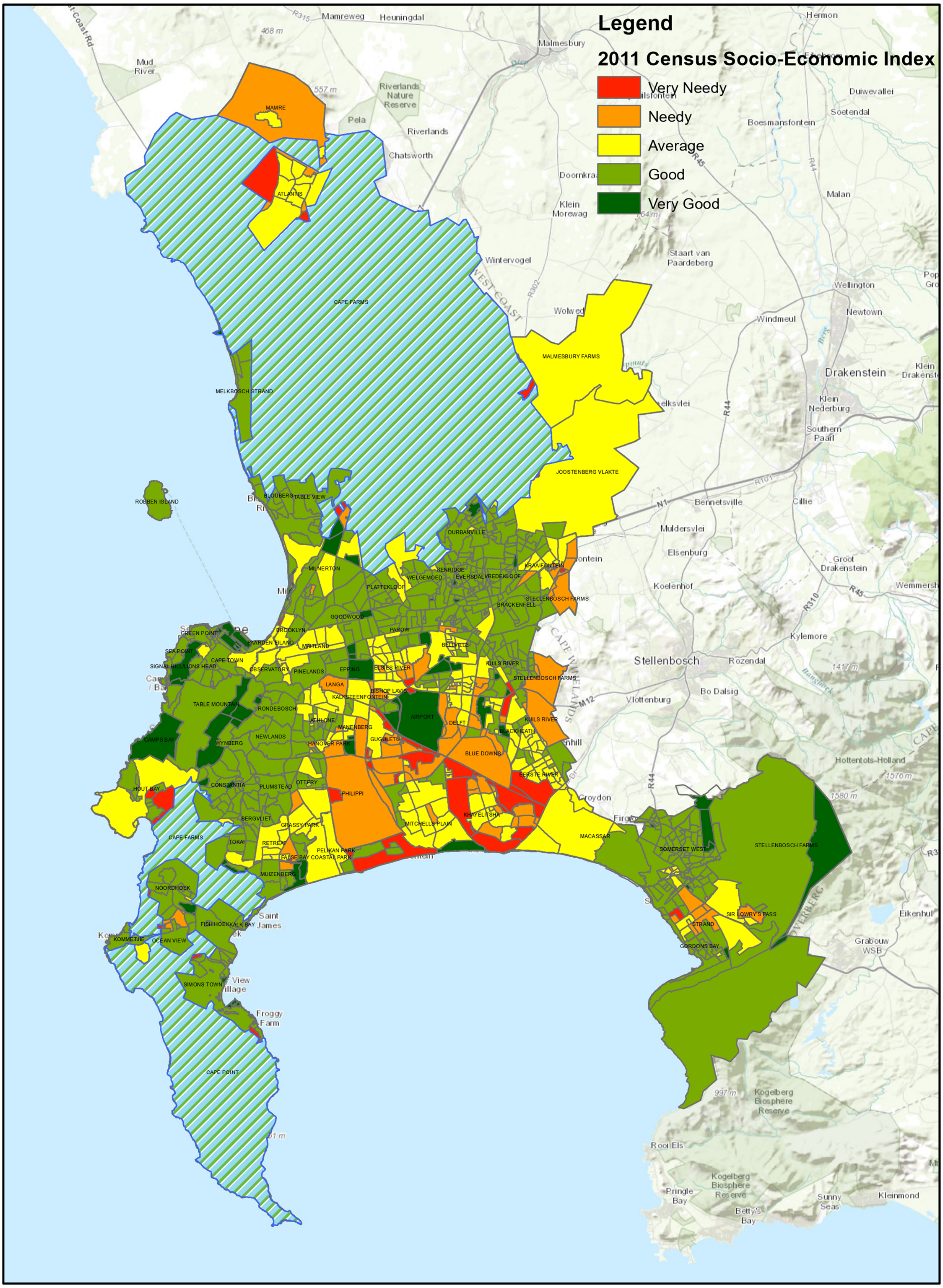

Supplementary Material (Figure S1): The Cape Town socioeconomic index map.
